# CRISPR Interference (CRISPRi) Inhibition of luxS Gene Expression in *E. coli*: An Approach to Inhibit Biofilm

**DOI:** 10.3389/fcimb.2017.00214

**Published:** 2017-05-26

**Authors:** Azna Zuberi, Lama Misba, Asad U. Khan

**Affiliations:** Medical Microbiology and Molecular Biology Lab., Interdisciplinary Biotechnology Unit, Aligarh Muslim UniversityAligarh, India

**Keywords:** biofilms, CRISPR, Cas9, Autoinducer-2, quorum sensing, luxS, knockdown

## Abstract

Biofilm is a sessile bacterial accretion embedded in self-producing matrix. It is the root cause of about 80% microbial infections in human. Among them, *E. coli* biofilms are most prevalent in medical devices associated nosocomial infections. The objective of this study was to inhibit biofilm formation by targeting gene involved in quorum sensing, one of the main mechanisms of biofilm formation. Hence we have introduced the CRISPRi, first time to target luxS gene. luxS is a synthase, involved in the synthesis of Autoinducer-2(AI-2), which in turn guides the initial stage of biofilm formation. To implement CRISPRi system for luxS gene suppression, we have synthesized complementary sgRNA to target gene sequence and co-expressed with dCas9, a mutated form of an endonuclease. Suppression of luxS expression was confirmed through qRT-PCR. The effect of luxS gene on biofilm inhibition was studied through crystal violet assay, XTT reduction assay and scanning electron microscopy. We conclude that CRISPRi system could be a potential strategy to inhibit bacterial biofilm through mechanism base approach.

## Introduction

Biofilm is the emergent form of bacterial life, described as the bacterial aggregates encased in self-producing matrix (Singh et al., 2000; Flemming et al., 2016) Bacteria in this type of sessile biofilm lifestyle develop resistance against the antimicrobial treatments (Whiteley et al., 2001). One such Gram-negative anaerobe is *E. coli*, known for its intestinal (InPEC) and extra intestinal (ExPEC) infections due to the formation of aggressive and dense bacterial biofilms. Mastitis, Urinary tract infections, Neonatal sepsis, enteric syndrome, Crohns disease and Hemorrhage are some of the reported infections in human, which are caused by *E. coli* (Vogeleer et al., 2014). Moreover, several medical implants associated infections such as prosthetic joints, grafts, shunts as well as intravascular and urinary catheters associated infections also come under the category of nosocomial infections caused by *E. coli* (Reisner et al., 2014). Genetically, each stage of biofilm formation is supplemented with the activation of different set of genes that controls the expression of its virulence factors. csgD, hha, bcsA operon, pgaC, fimB are some of the reported group of genes that are activated during the development of biofilm formation (Keseler et al., 2012). Maturation stage of biofilm is guided with production of auto-transporters (AidA and TibA) and Extracellular polymeric substance (EPS) which provide three dimensional characteristic structures to biofilms (Sharma et al., 2016), where quorum sensing (QS) plays a major role. QS is a density dependent chemical signaling method used by bacteria to control their collective behaviors like biofilm formation and pathogenesis (Keller and Surette, 2006). A number of genes (luxS, mqsR, qseB, qseC, pfs, flhD, fliA, motA, lsrK, lsrR, and csrA) are involved in quorum sensing mechanism of *E. coli*. luxS is a part of activated methyl cycle and involved in the production of Autoinducer-2 (AI-2) (Vendeville et al., 2005). The reports on luxS also revealed its AI-2 independent effects on biofilm formation where, about 23 genes were found to be influenced by luxS gene deletion in presence of glucose, while number increased to 63 in absence of glucose, but most of the genes were found to be involved in AI-2 synthesis, which in turn control quorum sensing (Wang et al., 2005).

CRISPR derived from bacterial immune system, it employs the use of genetic scissor, i.e., Cas9 endonuclease and two-component target identifying CRISPR-RNA duplex (crRNA and tracrRNA), the engineered chimeric form of which is called single guide RNA (sgRNA) (Jinek et al., 2012). The sgRNA is programmed to bind the target DNA by base pairing its complementary sequence adjacent to short DNA motif, called Protospacer adjacent motif (PAM). This PAM sequence is the only constraint with sgRNA binding as it is the mandatory requirement of Cas9. The PAM sequence merely depends on the species, from which the Cas9 derived. The most commonly used Cas9 is derived from *S. pyogenes* that uses NGG as PAM sequence (Jinek et al., 2012).

Apart from its editing potential, this technique also has its own role in regulating up (CRISPRa) and down (CRISPRi) (Lau, 2014) the gene expression, as the close proximity of DNA, RNA and protein in the form of Cas9-sgRNA complex acts as a scaffold to recruit the wide range of effectors and markers at specific DNA locations. CRISPRa and CRISPRi are two derivatives of CRISPR-Cas9 that works at transcriptional level and act as an alternate for gene regulation system (Lau, 2014).

The first glimpse of CRISPRi comes from the researchers at the Gladstone Institutes in San Francisco, California, who used this version of CRISPR gene editing to accurately and reversibly suppress gene expression in induced pluripotent stem cells (iPSCs) and derivative T cells and heart cells (Qi et al., 2013). Different from traditional genetic expression regulation that includes cutting down or inserting gene sequence, this technique tweak the gene expression by lodging its catalytically inactive or “dead” Cas9 at specific position, that in turn palpably control the gene expression by hindering transcriptional machinery to bind the DNA (Qi et al., 2013) The convalescent version of CRISPRi requires the attachment of 50 amino acids domain from transcription silencer called kruppel associated box (KRAB) to dCas9 that further prevents uncoiling of DNA for transcription and improves its efficiency (Lau, 2014).

In view of the above background we have initiated to knockdown luxS gene, which could be one of the approaches to control biofilm mediated infections. Hence, to our knowledge it is the first time we intended to use CRISPR (Clustered, Regularly Interspaced, Short, Palindromic, Repeat) derived CRISPR interference system to inhibit biofilm formation in *E. coli* by targeting quorum sensing gene (luxS).

## Materials and methods

### Bacterial culture

*E. coli* clinical strain (AK-117) having high potency of forming biofilm was used under this study. The strain was isolated from the urinary catheters of patient suffering from urinary tract infection (UTI) in Jawaharlal Nehru Medical College (JNMC), A.M.U, Aligarh, India. The microorganism was sub-cultured in Luria bertani (LB) broth, (Himedia labs, Mumbai, India). The plasmids named pdCas9 (expressing dCas9 endonuclease of *S. pyogenes*) and pgRNA (used for expressing gene specific sgRNA's) was commercially purchased from ADDGENE plasmids depository (plasmid #44249 and plasmid #44251) (Bikard et al., 2013) and sub-culture in LB media supplemented with proper antibiotic (ampicillin 100 μg/ml and chloramphenicol 25 μg/ml) and inducer (anhydrotetracycline, i.e., aTc, 2 μM), wherever needed. *E. coli* Top10 cells were used for transformation while the co-transformation was performed in AK-117. The bacteria were grown with (in broth) or without (on agar) shaking at 220 rpm at 37°C, overnight. To repress the gene, the knockdown strain was supplemented with 2 μM aTc with their respective antibiotics.

### Cloning of complementary sequences in pgRNA and creating knockdown strains

**Figure 1:**
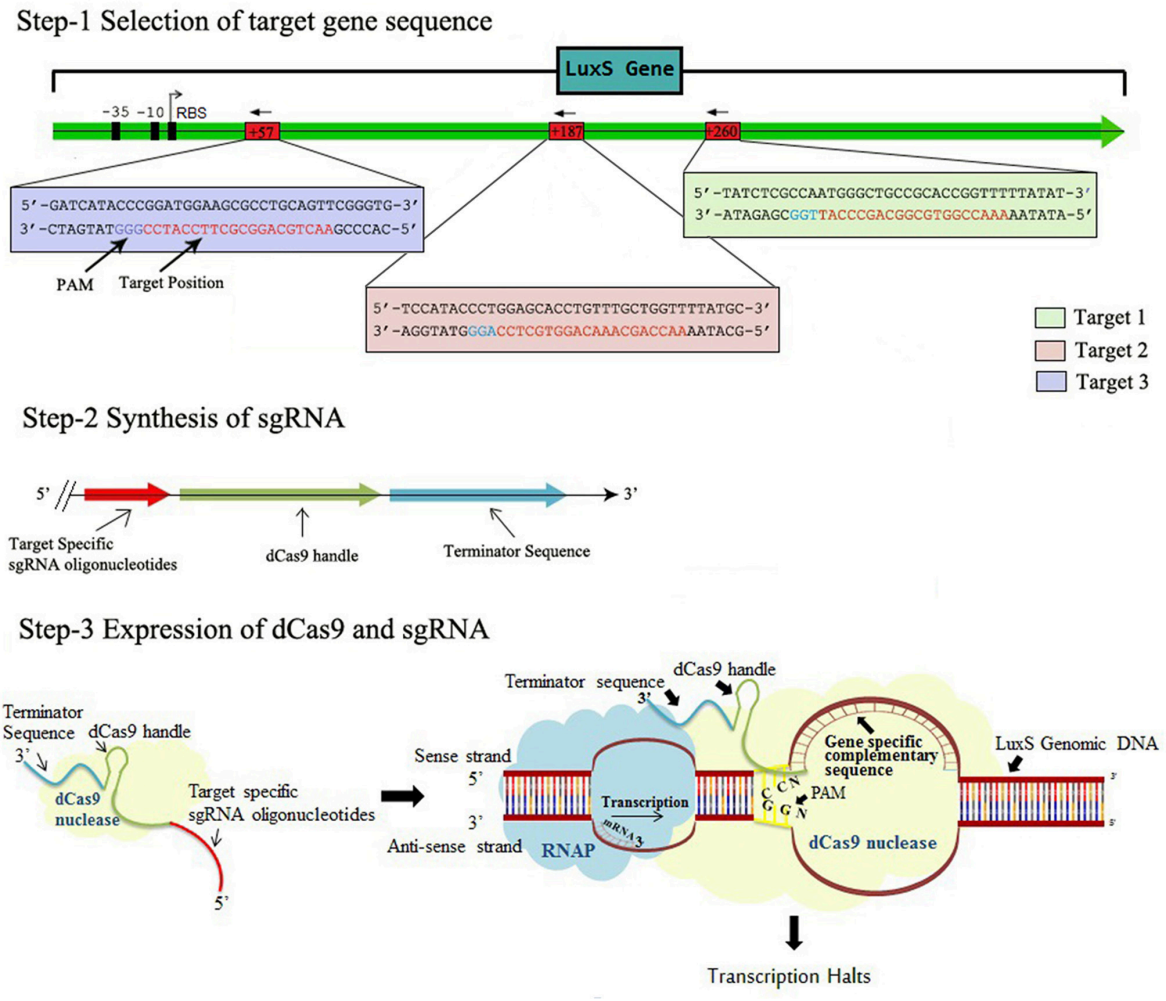
The Schematic representation of CRISPRi mechanism, Step-1 Selection of target gene sequence based on its distance from TSS side, PAM location, GC content, off-targeting. Step-2 Synthesis of sgRNA by appending dCas9 handle and terminating sequences. Step-3 Expression of dCas9 protein and sgRNA through cloning and co-transformation that finally blocks the transcription.

In order to express gene specific sgRNAs within the bacterial cell, the complementary sequences (20 bp region adjacent to PAM ie. immediately following to 5′-CCN-3′, Supplementary Table 1) to that gene was commercially synthesized in the form of primers also containing 35 nt part of the dCas9 handle (Figure 1, Steps 1 and 2). Inverse PCR was carried out to insert 20 bp region in the pgRNA. Total three sets of primers were synthesized to target luxS gene at different positions (Supplementary Table 2). Both forward and inverse primers were, first phosphorylated and inverse PCR was carried out using conditions mentioned in protocol (Larson et al., 2013) with the slight modification (Supplementary Table 3). Further the PCR products were purified using Gel extraction kit (Qiagen) yielding blunt ended linear fragments of approximately, 2.5 kb. The purified PCR products were cleaned from template DNA (pgRNA) using Dpn I and ligated using blunt end ligation kit, finally yielding circular plasmids bearing complementary region to that gene. The new sgRNA expressing plasmids were designated as pgRNA-LV1, pgRNA-LV2, pgRNA-LV3, pgRNA-LV4 and transformed in *E. coli* Top 10 competent cells. Single transformant from plate supplemented with proper antibiotic (ampicillin 100 μg/ml) was picked and colony PCR was performed using conditions mentioned in Supplementary Table 4. The PCR products were cleaned with exonuclease I and shrimp alkaline phosphatase and send to commercial services (Scigenome) for sequencing with L-F-colony primer (Supplementary Table 2).

The plasmids from confirmed clones were isolated and transformed into AK-117 strains along with pdCas9 to create the respective knockdown strains. The expression level of pdCas9 plasmid was already checked in AK-117.

### The expression of dCas9 protein

The expression profile of dCas9 protein was assayed through SDS-PAGE. The plasmid pdCas9 was transformed in AK-117 and transformants were picked as single colony from LB-agar plates (supplemented with 25 μg/ml of chloramphenicol) to give primary culture. Secondary culture was inoculated with 1% of primary culture and induced with 2 mM of aTc during exponential phase of bacterial growth. After 3–4 h of growth, the culture was centrifuged at 1,000 rpm (Sigma 12154 H) for 10 min at 4°C. The pellet obtained was dissolved in 4X SDS-dye along with 1 mol of DTT and heated at 95°C for 10 min. Finally, the samples containing bacterial protein as well as induced protein were loaded on SDS-PAGE gel.

### Extraction of total RNA

Trizol method was used to extract total RNA from respective knockdown strains grown in presence of inducer (2 μM aTc) and proper antibiotic concentration till log phase.

### RT-PCR and mRNA quantification

Bacterial total RNA was treated with RNase free DNase in order to remove DNA contamination, which was assessed after 30 cycles of PCR through ethidium bromide agrose gel analysis. Subsequently cDNA was prepared by high capacity cDNA Reverse Transcription Kits (applied biosystems, USA) according to manufacturer instruction. For quantification of mRNA, RT-PCR was performed using SYBR green PCR master mix, along with 150 ng of cDNA sample and appropriate primers (Supplementary Table 2). The cycle was carried out at 95°C for 10 min, 95°C for 15 s, 60°C for 30 s and finally 72°C for 30 s (Supplementary Table 6). The standard curves for respective transcripts were observed using 16s rRNA as control.

### Biofilm formation assay

The biofilm formation was assessed using protocol, reported earlier with the slight modification (Misba et al., 2016). An overnight culture of AK-117 (control) and knockdown strain was diluted to the ratio of 1: 250 in the fresh culture of Luria Bertani medium supplemented with (knockdown strains) or without (control) proper concentration of ampicillin (100 μg/ml), chloramphenicol (25 μg/ml) and 2 μM aTc. The 96 well U-shaped plates bearing culture (100 μl in each well) were kept at 37°C for 24 h without shaking. After incubation the media was removed carefully and the plate was gently rinsed with PBS (1x) solution to remove planktonic cells. Secondly, the biofilms in the wells were fixed using 37% formalin solution supplemented with 2% sodium acetate for 4–6 h at 4°C. Further each well was stained with 200 μl of 0.1% crystal violet solution at room temperature for 15–20 min and washed with PBS (1x) solution. The bound dye was released with 100 μl 95% absolute alcohol and the plates were kept at shaker for 5 min to release dye properly. Finally the biofilm formation was quantified by measuring optical density (O.D) of suspension at 630 nm with the help of microplate reader (BIORAD imark™ microplate reader India).

### XTT reduction assay

To assess cellular viability and metabolic activity XTT assay was carried out as described earlier (Misba et al., 2016). XTT was dissolved in filter sterilized PBS solution to a concentration 1 mg/ml and stored at −80°C till used. Menadione of concentration 0.4 mM was also freshly prepared in acetone before each assay. Fresh mixture of 20:1 volume of XTT and menadione was used. After 24 h of growth, adherent biofilms were washed with 200 μl PBS solution to remove planktonic cells. After this 42 μl of XTT and menadione solution along with 158 μl of PBS solution was dispensed in each well and kept at 37°C in dark for 4 h. The calometric change was measured after 4 h using microtiter plate reader at 490 nm. The intensity of orange color formazan compound was measured, that quantify the ability of metabolically active sessile cells to reduce tetrazolium salt (2,3-bis(2-methoxy-4-nitro-5-sulfo-phenyl)-2H-tetrazolium-5-carboxanilide).

### Scanning electron microscopy

The biofilm was grown in six well microtiter plate on coverslips of treated (knockdown) and control bacterial cells. After 24 h of growth, biofilm was fixed using 2.5% gluteraldehyde and 2% formaldehyde for 2 h at 4°C followed by dehydration with increasing concentration of ethanol (20, 40, 60, 80 and 100%). After drying, the coverslips were mounted and sputter coated with gold-palladium to be analyzed through Scanning Electron Microscope (SEM).

### Statistical analysis

The results were represented as mean ± standard deviation. Each experiment was performed in triplicate and compared with control for analyzing student *t-test*, two-tailed hypothesis, (^*^P < 0.05, *t*-test, two sided), (^**^P < 0.005, *t*-test, two sided). One way analysis of variance (ANOVA) and free online software (http://www.physics.csbsju.edu/stats/anova.html), was used for comparison. *P* < 0.05 were considered statistically significant.

## Results

### Construction of plasmid to express sgRNA in bacterial cells

**Figure 2:**
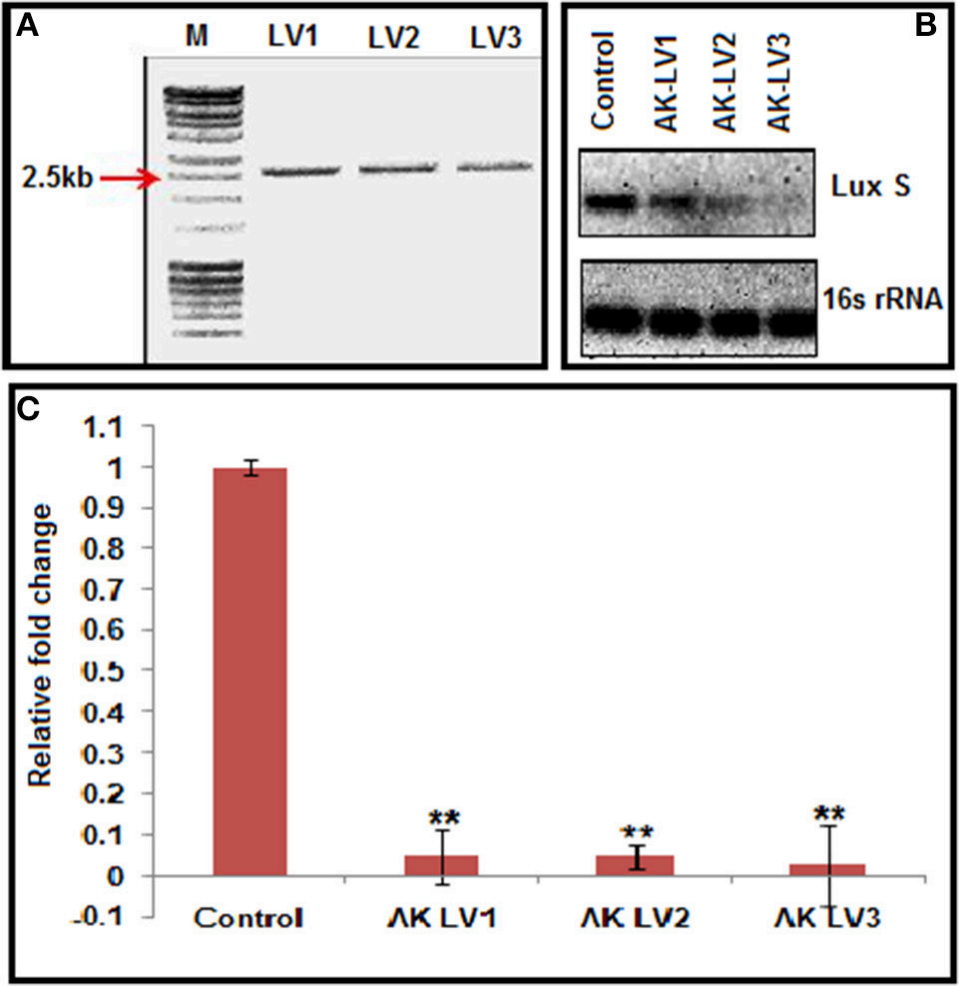
**(A)** Inverse PCR product of 2,500 bp showing successful integration of target specific sgRNA. **(B)** Semi-quantitative data of LuxS gene (Control, AK-LVI, AK-LV2, and AK-LV3) and 16s rRNA gene as positive control. **(C)** Expression profile of luxS gene evaluated through qRT-PCR after CRISPRi mediated inhibition, (^**^P < 0.005, *t*-test, 2 sided).

CRISPRi was designed to knockdown the luxS, target specific sgRNA expressing plasmids were constructed using pgRNA through inverse PCR (primers LV1-F LV2-F LV3-F and L-R Supplementary Table 2). Successful inverse PCR results were confirmed by 2.5 kb product through gel electrophoresis (Figure 2A). The PCR products were purified, ligated and transformed in TOP 10, competent *E. coli* cells and the data was confirmed through sequencing of linearized DNA fragments containing target specific sgRNA, generated through colony PCR with different set of primers (Supplementary Table 2). The new sgRNA expressing plasmids were designated as pgRNA-LV1, pgRNA-LV2 and pgRNA-LV3.

### Expression of dCas9 nuclease in AK-117

SDS-PAGE profile of control as well as induced sample of protein is clearly shown in Supplementary Figure 1. The dark band corresponding to ~160 kDa in Lane 3 shows successful expression of pdCas9 protein in AK-117 strain.

### CRISPRi induced luxS repression

Each of the plasmid expressing different luxS sgRNA variants (pgRNA-LV1, pgRNA-LV2 and pgRNA-LV3), was co-transformed with pdCas9 in AK-117 separately to create respective knockdown strains (AK-LV1, AK-LV2 and AK-LV3). The efficacy of CRISPRi in these strains was observed through semi quantitative estimation by ethidium bromide stained gel, using regular PCR (Supplementary Table 5) and through relative qRT-PCR taking 16s rRNA gene as an endogenous control for normalization (Supplementary Table 6). The varying density of band itself indicated the differential expression of gene transcripts in respective luxS knockdown strains. Being a endogenous control, 16s rRNA band density remains same in all variants (Figure 2B). The relative qRT-PCR data of knockdown strains also revealed the dramatic decrease in the expression of luxS gene. Taking control as 1, the values of luxS gene expression reported for three variants AK-LV1, AK-LV2, AK-LV1 are 0.04792, 0.04702, and 0.02709 respectively (Figure 2C). The *p*-values for all three variants were obtained < 0.005.

### Quantification of biofilm formation

**Figure 3:**
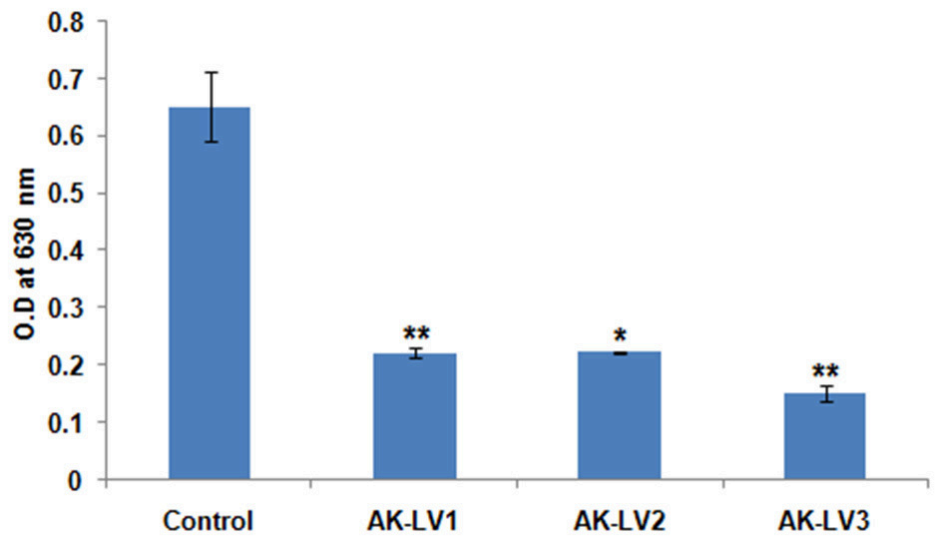
Effect on biofilm formation of control and knockdown strains (AK-LV1, AK-LV2, and AK-LV3). The data represents an average of triplicate experiments ± S. (^*^P < 0.05, *t*-test, 2 sided), (^**^P < 0.005, *t*-test, 2 sided).

luxS gene is directly involved in biofilm formation through quorum sensing mechanism. To inhibit the biofilm production, luxS gene was targeted through CRISPRi in AK-117. The biofilm forming tendency of AK-117 was preliminary investigated through crystal violet assay. Down regulation of luxS gene in AK-LV1, AK-LV2, and AK-LV3 led to the decrease in biofilm formation in these respective knockdown strains, which was quantified by crystal violet assay. The significant reduction in biofilm formation was noticed in AK-LV3 followed by AK-LV2 and AK-LV1 (Figure 3).

### Cell viability assay

**Figure 4:**
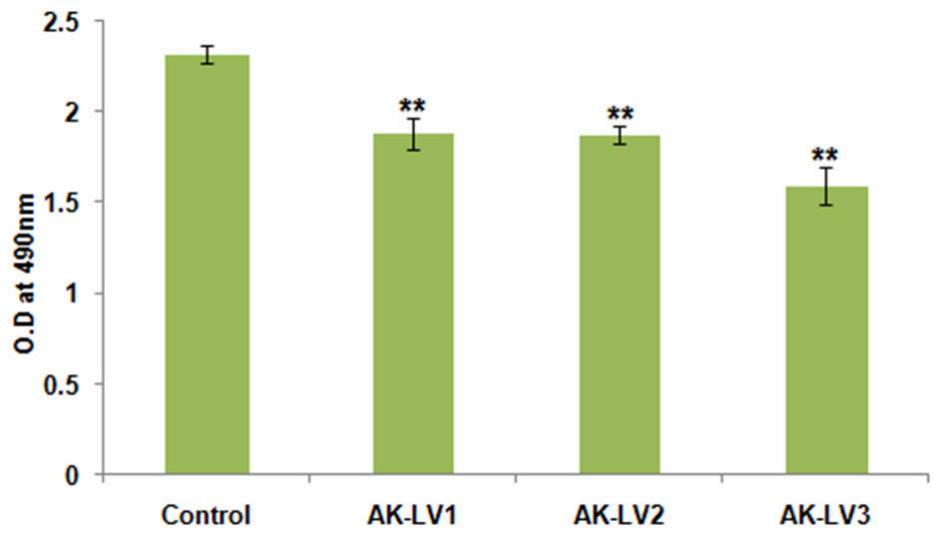
Effect on cell viability and metabolic activity. The data represents an average of triplicate experiments ± SD. (^**^P < 0.005, *t*-test, 2 sided).

To test the metabolic activity and viability of created knockdown strains the converted amount of XTT was measured. The viability of AK-117 derived luxS knockdown strains (AK-LV1, AK-LV2, and AK-LV3) was recorded as 82, 81, and 69% respectively (Figure 4).

### Effect of CRISPRi on biofilm formation

**Figure 5:**
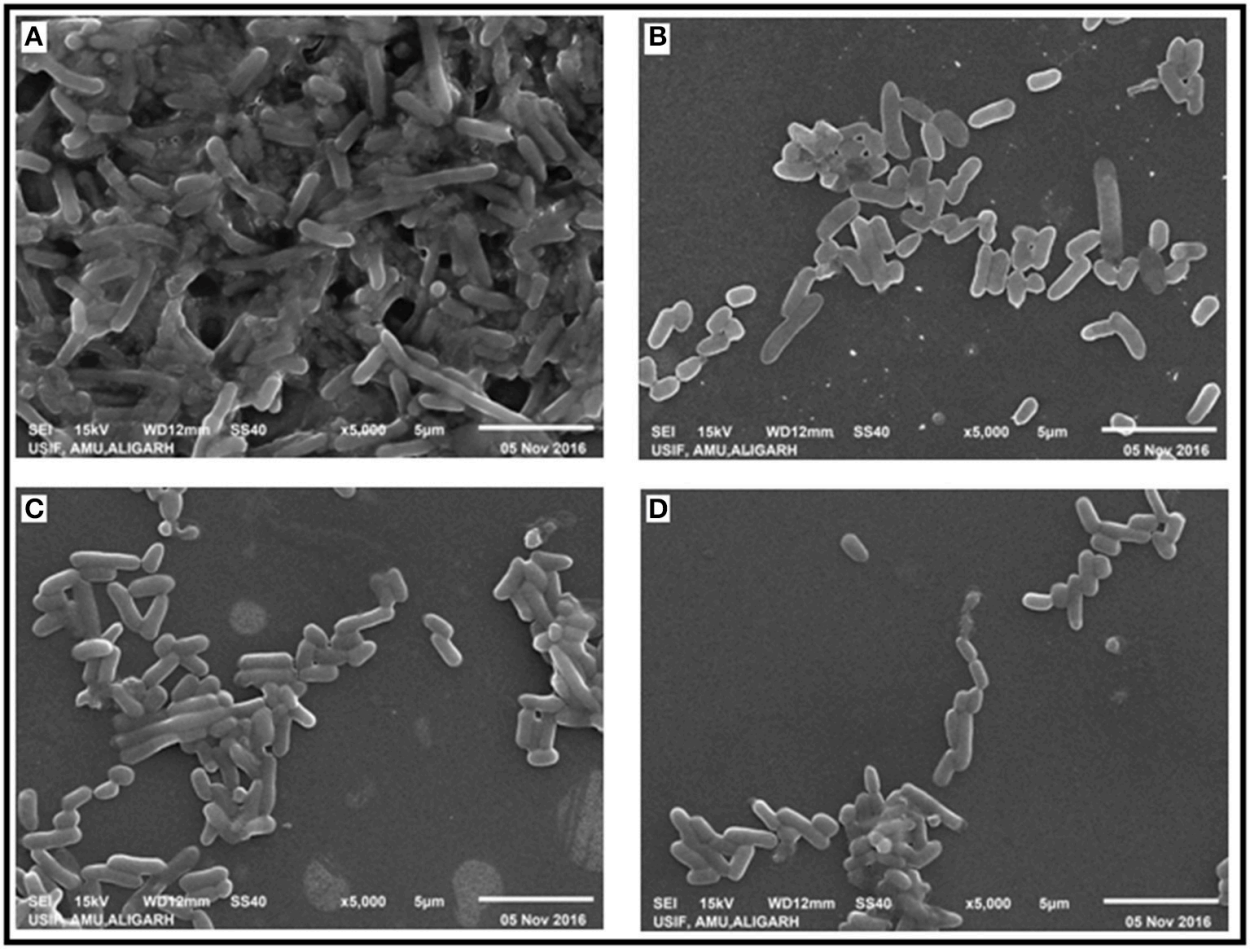
Effect of luxS down regulation on biofilm architecture. SEM images **(A)** AK-117 biofilm as control **(B)** AK-LV1 **(C)** AK-LV2 **(D)** AK-LV3.

The effect of CRISPRi mediated luxS gene silencing on AK-117 biofilms was studied using Scanning Electron Microscopy (SEM). The cells of control sample were seen embedded in their self-producing matrix while treated samples had discrete cell colonies. The AK-LV3 variant had shown maximum inhibition in biofilm formation, while least was recorded in AK-LV1 (Figure 5).

## Discussion

Biofilm is the root cause of about 80% of human microbial infections that are chronic and recurrent in nature (Costerton et al., 1999). Broadly these infections are categorized in two types; indwelling medical devices associated infections and host tissues native biofilm infections (Donlan, 2001; Burmølle et al., 2010). One such Gram-negative anaerobe known for its biofilm forming tendency is *E. coli*. It is generally involved in infections associated with indwelling medical devices like implants, grafts, prosthetic joints, shunts, intravascular and urinary catheters (Reisner et al., 2014). In this study we have introduced CRISPRi, a new gene targeting strategy to inhibit biofilm formation. This is the first time we came out with the concept of CRISPRi for bacterial biofilm inhibition. CRISPRi inhibition is well known for its appreciated results in gene regulation, higher precision, high throughput and a special advantage over “knock-out” screens as it creates different levels of targeted knockdown which helps to study behavioral changes in cell when a gene is expressed at varying levels.

As quorum sensing is thought to be one of the major mechanisms involved in biofilm formation, hence, we planned to inhibit biofilm formation by targeting genes that are directly involved in quorum sensing. Among, luxS, mqsR, qseB, qseC, pfs, flhD, fliA, motA, lsrK, lsrR, and csrA genes, we initiated our study to target luxS, a synthase involved in the synthesis of AI-2 (a furanosyl borate di-ester, and a member of a signaling molecules used in quorum sensing) (Schauder et al., 2001) that in turn effects early phase of biofilm, its architecture and its mass as reported earlier (Barrios et al., 2006).

To set the platform of our study, we constructed the plasmid, expressing target specific sgRNA. Three different nucleotide positions on the gene were targeted (Figure 1). The purpose of taking three different targets was to evaluate the CRISPRi mediated inhibition separately in each variant as well as to compare the effect in all variants. We also checked the expression of *S. pyogens* derived dCas9 nuclease in strain AK-117. For that we commercially purchased pdCas9 plasmid from ADDGENE laboratory and checked its expression level in AK-117 (Supplementary Figure 1). To further implement our studies, we co-transformed pdCas9 along with sgRNA expressing plasmids. Three knockdown variants were obtained whose gene expression was checked by qRT-PCR. The maximum suppression was noticed in AK-LV3 and minimum in AK-LV1. The proposed reason for such variation in gene expression level may be due to different target sequences, its distance from TSS (Transcription start site), transcription factor binding and chromatin states at the target sites (Radzisheuskaya et al., 2016).

Perusing the work, the effect of luxS on biofilm was evaluated through Crystal violet. The dramatic decrease in biofilm formation was observed in AK-LV3 followed by AK-LV2 and AK-LV1. The results found consistent with luxS gene expression level. The XTT assay was also performed to observe cell viability and its metabolic activity. The data suggested most of the cell were found metabolically active but not able to form biofilm. The reason may be due to the reduce EPS production or inhibition in production of signaling molecules (AI-2) that are responsible for cell to cell communication and may guide the initial stage of biofilm formation. The morphological changes in biofilm architecture were also visualized by SEM. Large cellular aggregates embedded in a self-producing matrix was observed in control while discrete cells having small clumps were observed in knockdown strains, which further justified the role of luxS mediated quorum sensing in biofilm formation.

## Conclusion

This is the first report on CRISPRi mediated inhibition in bacterial biofilms. The study was conducted by targeting luxS gene, causing inhibition of biofilm formation through intervention of quorum sensing mechanism. Hence we can propose this as a potential approach to inhibit biofilm of bacterial population in nosocomial or environmental settings through direct delivering the CRISPRi edited cells at localized bacterial cells by nucleic acid conjugation.

## Author contributions

AK designed experiments, analyzed data and wrote the manuscript. AZ performed experiments, and wrote the manuscript, LM performed experiments.

### Conflict of interest statement

The authors declare that the research was conducted in the absence of any commercial or financial relationships that could be construed as a potential conflict of interest.
