# Supplementary material for: CRISPR Interference (CRISPRi) Inhibition of luxS Gene Expression in E. coli: An Approach to Inhibit Biofilm
Source: Front Cell Infect Microbiol. 2017 May 26;7:214. doi: 10.3389/fcimb.2017.00214 (PMC5445563; doi:10.3389/fcimb.2017.00214)
Supplement: Supplementary file 1 [file DataSheet1.PDF]

CRISPR interference (CRISPRi) induced inhibition of luxS gene in *E. coli*: an approach to inhibit biofilm.

SUPPLEMENTARY INFORMATION

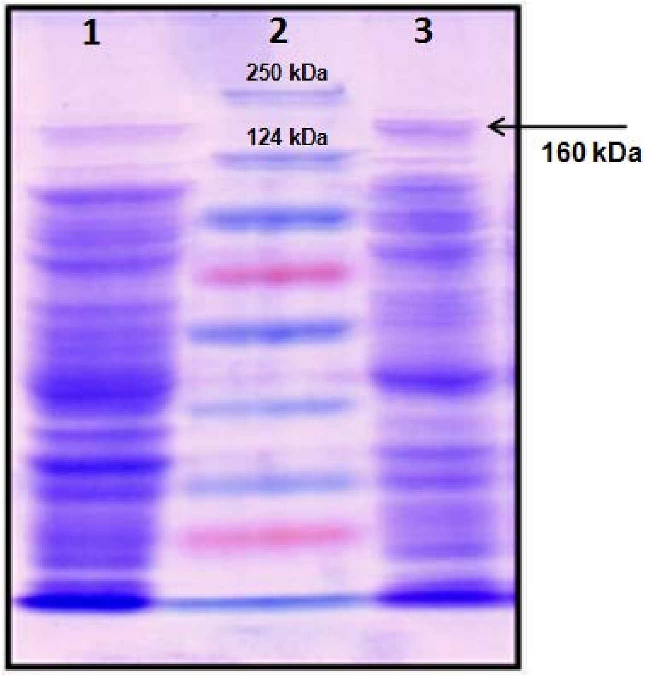

Supplementary Figure 1.SDS-PAGE gel showing expression of dCas9 protein. Lane 1: AK-117 (control), Lane 2: Protein marker, Lane 3: AK-117 with induced pdCas9.

Supplementary Table 1.sgRNA sequences used in the study

S.No	sgRNA sequence	Gene name	Gene synonym	Distance to TSS	Location	Efficacy Score (E)	Specificity Score (S)	E+S Score
1	AAACCGGTGCGGCAGCCCAT	luxS	ECK2681	+260	2814473	20	0	20
2	AACCAGCAAACAGGTGCTCC	luxS	ECK2681	+187	2814546	20	0	20
3	AACTGCAGGCGCTTCCATCC	luxS	ECK2681	+57	2814676	20	0	20

Supplementary Table 2.Primers used in the study

NAME	SEQUENCE (5’-3’)	PURPOSE
LV1-F	AAACCGGTGCGGCAGCCCATGTTTTAGAGCTAGAAATAGCAAGTTAAAATAAGGC	Forward primer used to Insert gene targeting sequence in pgRNA.
LV2-F	AACCAGCAAACAGGTGCTCCGTTTTAGAGCTAGAAATAGCAAGTTAAAATAAGGC	Forward primer used to Insert gene targeting sequence in pgRNA.
LV3-F	AACTGCAGGCGCTTCCATCCGTTTTAGAGCTAGAAATAGCAAGTTAAAATAAGGC	Forward primer used to Insert gene targeting sequence in pgRNA.
L-R	ACTAGTATTATACCTAGGACTGAGCTAGC	Reverse primer used to Insert gene targeting sequence in pgRNA
L-F-colony	GGGTTATTGTCTCATGAGCGGATACATATTTG	Forward primer for colony PCR
L-R-colony	CGCGGCCTTTTTACGGTTC	Reverse primer for colony PCR
L-F-rt	GTGTTCGATCTGCGCTTCTG	Forward primer for RT-PCR
L-R-rt	GGATCCCTCTTTCTGGCATCA	Reverse primer for RT-PCR

Supplementary Table 3.Conditions used for Inverse PCR.

CYCLE NUMBER	DENATURING	ANNEALING	EXTENTION
1	98□C, 30s		
2-26	98□C, 10s	62□C, 30s	72□C, 1 min
27			72□C, 5 min

Supplementary Table 4.Conditions used for Colony PCR.

CYCLE NUMBER	DENATURING	ANNEALING	EXTENTION
1	95□C, 3 min		

2-31	95□C, 30s	62□C, 30s	72□C, 2 min
32			72□C, 5 min

Supplementary Table 5.Conditions used for PCR (Semi quantitative estimation).

CYCLE NUMBER	DENATURING	ANNEALING	EXTENTION
1	95□C, 5 min		
2-29	95□C, 15 s	60□C, 30s	72□C, 30 s
30			72□C, 7 min

Supplementary Table 6.Conditions used for qRT-PCR.

CYCLE NUMBER	DENATURING	ANNEALING	EXTENTION
1	95□C, 10 min		
2-39	95□C, 15 s	60□C, 30s	72□C, 30 sec
40			72□C, 7 min
